# Feasibility of vagus nerve modulation (VNMM/taVNS) in cognitive-swallowing dual-task paradigms: a fNIRS pilot study

**DOI:** 10.3389/fnins.2025.1619532

**Published:** 2025-08-11

**Authors:** Zhiyong Wang, Keling Cheng, Junhui Bai, Cuicui Zhang, Jun Ni

**Affiliations:** ^1^Department of Rehabilitation, The First Affiliated Hospital, Fujian Medical University, Fuzhou, China; ^2^Department of Rehabilitation, National Regional Medical Center, Binhai Campus of the First Affiliated Hospital, Fujian Medical University, Fuzhou, China

**Keywords:** transauricular vagus nerve stimulation (taVNS), vagus nerve magnetic modulation, functional near-infrared spectroscopy (fNIRS), cortical excitability, brain connectivity, feasibility study

## Abstract

**Background:**

Non-invasive vagus nerve stimulation techniques show promise for modulating cortical networks, but their comparative effects during combined cognitive-swallowing tasks remain underexplored.

**Aims:**

This feasibility study aimed to: (1) establish a protocol for assessing transauricular vagus nerve stimulation (taVNS) and VNMM effects using fNIRS during dual-task paradigms, and (2) compare their impacts on cortical activation and functional connectivity.

**Methods:**

This protocol (ChiCTR2200065698) consisted of two separate blocks, a cognitive task (*n* = 25) and a swallowing task (*n* = 25), and healthy subjects in either block were randomly assigned to the taVNS and VNMM groups. The subjects underwent swallowing or cognitive task-state data acquisition before and after the trial intervention. The functional near-infrared spectroscopy (fNIRS) data were analyzed by generalized linear modeling (GLM) and seed-based correlation analysis to assess the cortical excitability and brain connectivity, and a BrainNet Viewer was used to visualize the intervention effects.

**Results:**

Under the cognitive and swallowing task-state fNIRS protocols, both taVNS and VNMM significantly enhanced the activation effects and intra/inter-hemispheric brain network connectivity in the cognitive or swallowing-related brain regions (*p* < 0.05).

**Conclusions:**

This study demonstrates the feasibility of using fNIRS to differentiate taVNS/VNMM effects during dual-task paradigms. Preliminary data suggest VNMM may offer superior network modulation, warranting larger trials to validate behavioral correlates.

**Clinical trial registration:**

Chictr.org.cn, identifier: ChiCTR2200065698.

## 1 Introduction

Transcutaneous auricular vagus nerve electrical stimulation (taVNS) is an emerging, non-invasive, adjuvant therapy that appears to modulate brain physiology through electrical stimulation of the auricular branch of the vagus nerve. Vagus nerve magnetic modulation (VNMM) is extracranial vagus nerve stimulation using repetitive transcranial magnetic stimulation (Use of rTMS on the mastoid, which is the body surface meridian of the auricular branch of the vagus nerve). These two techniques can also be collectively referred to as non-invasive vagus nerve modulation techniques (VNM). VNM can be a novel and promising therapeutic modality to promote neurological recovery in stroke patients. According to existing studies, VNM can effectively improve upper limb dysfunction in acute, subacute, and chronic stroke, and the potential mechanisms of neuroprotection mediated by VNM include anti-inflammatory pathways, anti-apoptosis and autophagy, promotion of neural and synaptic plasticity, and protection of blood-brain barrier integrity (Ahmed et al., 2022; Ananda et al., 2023). As the exploration related to this technology in the field of post-stroke upper limb physical dysfunction continues to deepen, the possibility of taVNS and VNMM in promoting the improvement of post-stroke cognitive dysfunction and swallowing dysfunction has also been proposed in recent years (Cheng K. et al., 2022; Wang Y. et al., 2022).

Swallowing is a complex process involving multiple systems (cognitive, sensory, and motor, among others) in its regulation, involving the involvement of multiple centers and the coordination of multiple muscle groups in order to push food or liquids from the oral cavity to the stomach, while protecting the airway and minimizing residue (Pitts and Iceman, 2023). Swallowing movements in normal humans can be divided into five phases: preoral, oral preparatory, oral, pharyngeal, and esophageal phases. Different factors (neurologic control, myogenic and neuromuscular junction, etc.) may lead to different degrees of impairment of swallowing function at each stage, with different manifestations of dysphagia (Sasegbon et al., 2025; Thiyagalingam et al., 2021). Among them, post-stroke dysphagia is more common, and some studies have reported that the prevalence of dysphagia in stroke patients is as high as 43%−83%, which significantly increases the risk of complications such as aspiration pneumonia, poor nutritional status, and hydroelectrolytic disorders in patients recovering from stroke (Hurtte et al., 2023; Rajati et al., 2022).

It is worth noting that performing a complete swallowing process requires good cognitive functions, such as attention, visuospatial, and executive functions, etc. Impairment of different cognitive components affects swallowing function and training effects, which may be related to dysfunctions in the patient's early swallowing activity in terms of perception of food, ingestion procedures, and natriuretic maneuvers (Dehaghani et al., 2021; Nagatani et al., 2023). Human swallowing activity is regulated by multiple layers of complex regulation in the cortical swallowing center, subcortical fibers, and the brainstem swallowing center, of which the main role is to control the initiation and oropharyngeal phase (Bhutada et al., 2022). brainstem swallowing centers, of which the main role of cortical swallowing centers is to control swallowing initiation and oropharyngeal phases, mainly including cortical motor and cortical sensory centers and prefrontal areas (Qin et al., 2023; Wei et al., 2024). Cognition is associated with prefrontal, parietal, and occipital regions of the cerebral cortex, in which the prefrontal cortex is a key region for cognitive functions, playing a key role in decision-making, planning, attention, working memory, and inhibitory control. Meanwhile, it has been found that the prefrontal lobe is also involved in the swallowing process, and damage to the prefrontal lobe not only affects cognitive function, but may also lead to swallowing disorders during the oropharyngeal phase (Gong et al., 2025). Therefore, it is crucial to modulate the cortical centers shared by cognitive and swallowing functions. In view of this, the present study used the Functional Near-Infrared Spectroscopy Neuroimaging (fNIRS) technique to observe possible changes in excitability and connectivity of cortical centers in cognitive and swallowing task paradigms in healthy subjects in the context of taVNS and VNMM intervention-mediated settings.

Both taVNS and VNMM are neuromodulation techniques that intervene at the vagal nerve meridian by mediating peripheral pathways. And swallowing and cognitive functions have shared central targets in the cerebral cortex, but the potential mechanisms and effect differences between taVNS and VNMM in promoting cognitive and swallowing improvements are not clear. The aim of this study was to explore the mediating role of swallowing and cognition, and to observe whether taVNS and VNMM techniques could simultaneously affect cognitive and swallowing-related cortical brain region functions. Therefore, we propose to conduct a double-blind randomized controlled trial using the fNIRS technique (swallowing task + cognitive task) in healthy individuals, so as to assess whether there are overlapping effects of the two measures at the level of cortical excitability and connectivity, as well as specificity, and to provide additional evidence for clinical application and practice.

## 2 Materials and methods

### 2.1 Study design

This was a double-blind, two-group randomized controlled trial comparing the effects of taVNS and VNMM on cortical excitability in healthy subjects. This protocol involved two separate blocks of cognitive and swallowing tasks, with healthy subjects in each block randomly assigned to the taVNS and VNMM groups. In the VNMM group, the mylohyoid motor-evoked potential (MH-MEP) was measured prior to the intervention to determine the intensity of the magnetic stimulation output. All subjects underwent functional near-infrared spectroscopy (fNIRS) neuroimaging data acquisition before and after the trial intervention. The study protocol was approved by the local Ethics Committee of the First Affiliated Hospital of Fujian Medical University (approval number: MRCTA, ECFAH of FMU [2021] 605). The study was preregistered at Chictr.org.cn (ChiCTR2200065698). All research procedures were conducted in accordance with Good Clinical Practice, the Declaration of Helsinki, and Ethical Review Methods for Biomedical Research on Human Beings.

### 2.2 Participant recruitment and grouping

**Figure 1:**
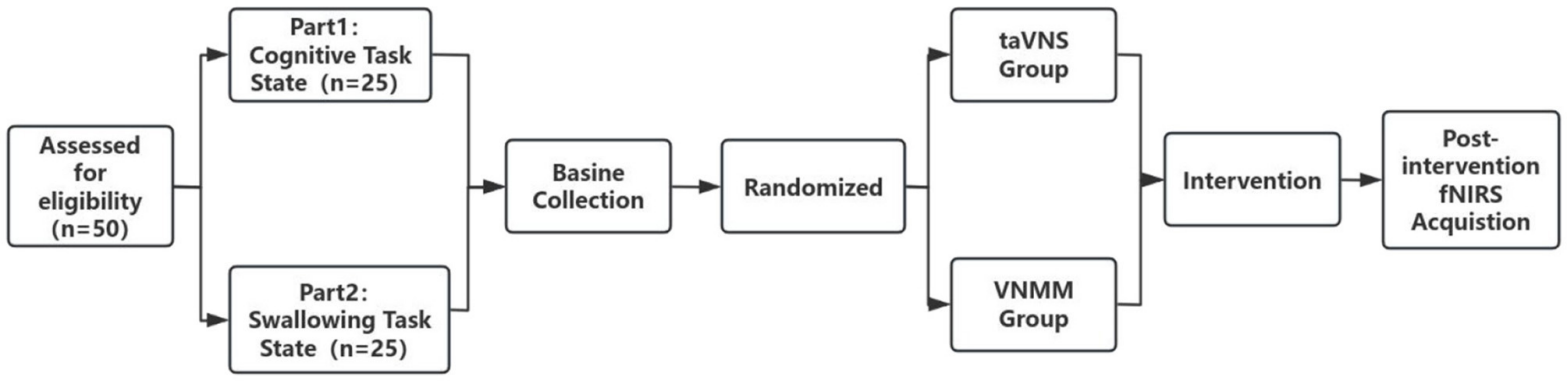
Flow chart of experimental design.

For this randomized controlled trial, 50 healthy subjects were recruited from Fujian Hospital, an affiliate of Huashan Hospital, Fudan University. Twenty-five participants underwent cognitive task-state fNIRS data collection, and 25 participants underwent swallowing task-state fNIRS testing (Figure 1). The inclusion criteria were (1) aged between 20 and 40; (2) clear-headed, stable vital signs; (3) able to understand and execute physician instructions; and (4) signed written informed consent prior to the study. The exclusion criteria included (1) serious medical conditions; (2) epileptic seizure history; (3) pregnancy or breastfeeding; and (4) intracranial metal implantation and tumors.

A blinded evaluator performed baseline assessments for all the participants. Allocation assignments were placed in sequentially numbered, opaque, and sealed envelopes by an independent researcher not involved in the study. The envelopes were handed over to the experimental operator following the completion of the allocation. After the baseline assessment, the subjects unsealed the envelopes in the order of recruitment. All subjects were randomly assigned to the taVNS and VNMM groups, then received the corresponding interventions. Cognitive or swallowing fNIRS protocol data were collected for all the subjects immediately after the intervention.

### 2.3 Vagus nerve magnetic modulation

**Figure 2:**
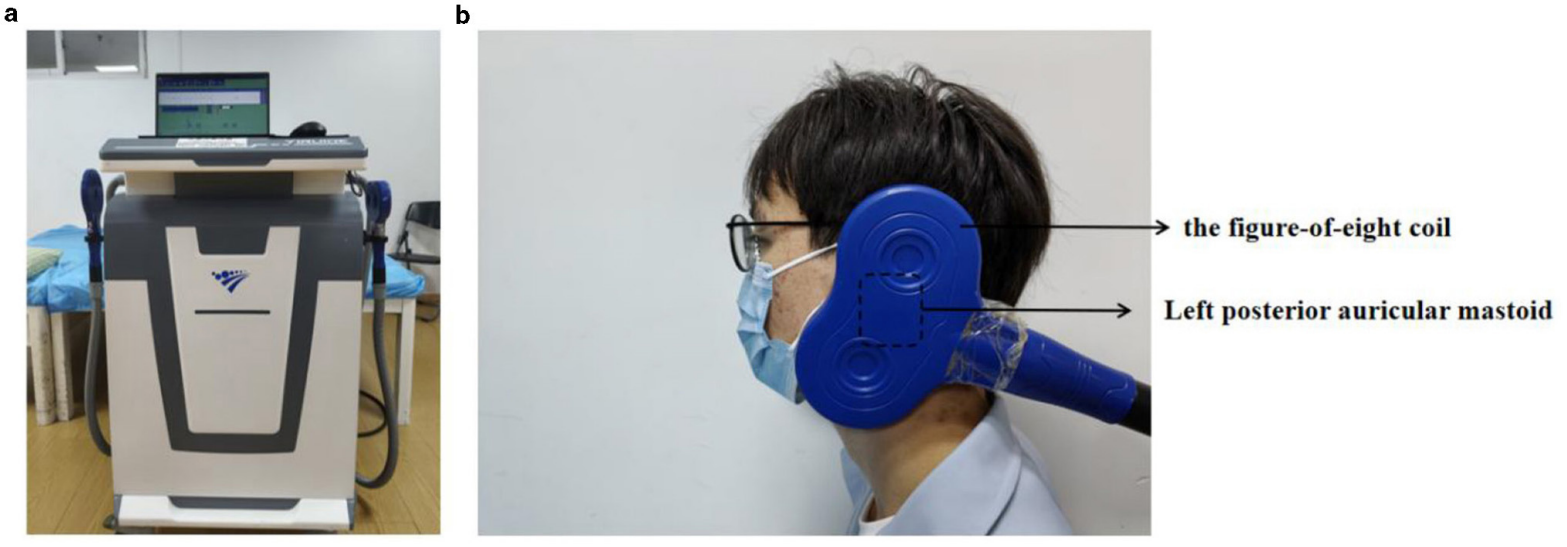
**(a)** VNMM equipment. **(b)** Schematic diagram of coil position for VNMM.

A transcranial magnetic device (Model CCY-II; Wuhan Yiruide Medical Equipment Co., Ltd., Wuhan, China, YZB-20142211249) was configured with a figure-of-eight coil positioned tangentially to the left mastoid (Wang et al., 2022a; Lin et al., 2018) (the location of the vagus nerve through the jugular foramina at the cranium) (Figure 2). The coil output intensity was 90–110% of the resting motor threshold (rMT) as the stimulus intensity based on the assessment of MH-MEP. Lying at the right lateral position, each subject received the 20-min intervention at an excitatory frequency of 5 Hz for a total of 1,200 pulses (Wang et al., 2022a; Lin et al., 2018).

### 2.4 Transauricular vagus nerve stimulation

**Figure 3:**
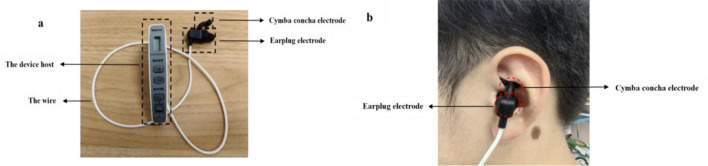
**(a)** Components of taVNS equipment. **(b)** Schematic diagram of wearing taVNS device.

This study used an auricular vagus nerve stimulator (Model: TENS-200A, Suzhou, China) designed by the Acupuncture and Moxibustion Research Institute of the Chinese Academy of Traditional Chinese Medicine. This instrument contains two parts: the host body and ear electrode (an earplug electrode and auricle electrode) (Figure 3a). Prior to the equipment operation, the left ear of the subjects in the taVNS group was sterilized with 75% alcohol, which facilitated the improvement of the efficacy of the intervention. In light of safety and efficacy considerations, the left cymba conchae was chosen as the site of stimulation since vagal efferent fibers leading in the direction of the heart are usually located on the right side. The cymba concha electrode (5.8 × 12 mm) was fitted to the innervation area of the auricular branch of the vagus nerve, and the cymba concha electrode (9.8 × 12 mm) was tightly attached to the external auditory canal (Figure 3b).

The parameters were set as a combination of a stimulation frequency of 20 Hz for 7 s and 4 Hz for 3 s, both of which were alternated, with an output pulse width of 0.2 ms. The intensity of the taVNS stimulation was a sub-threshold current of pain that the subjects could tolerate without sharp nociception, typically 5–10 mA, and the total duration of the intervention was 20 min (Colzato et al., 2018; Wang et al., 2022b).

### 2.5 Functional near-infrared spectroscopy neuroimaging

**Figure 4:**
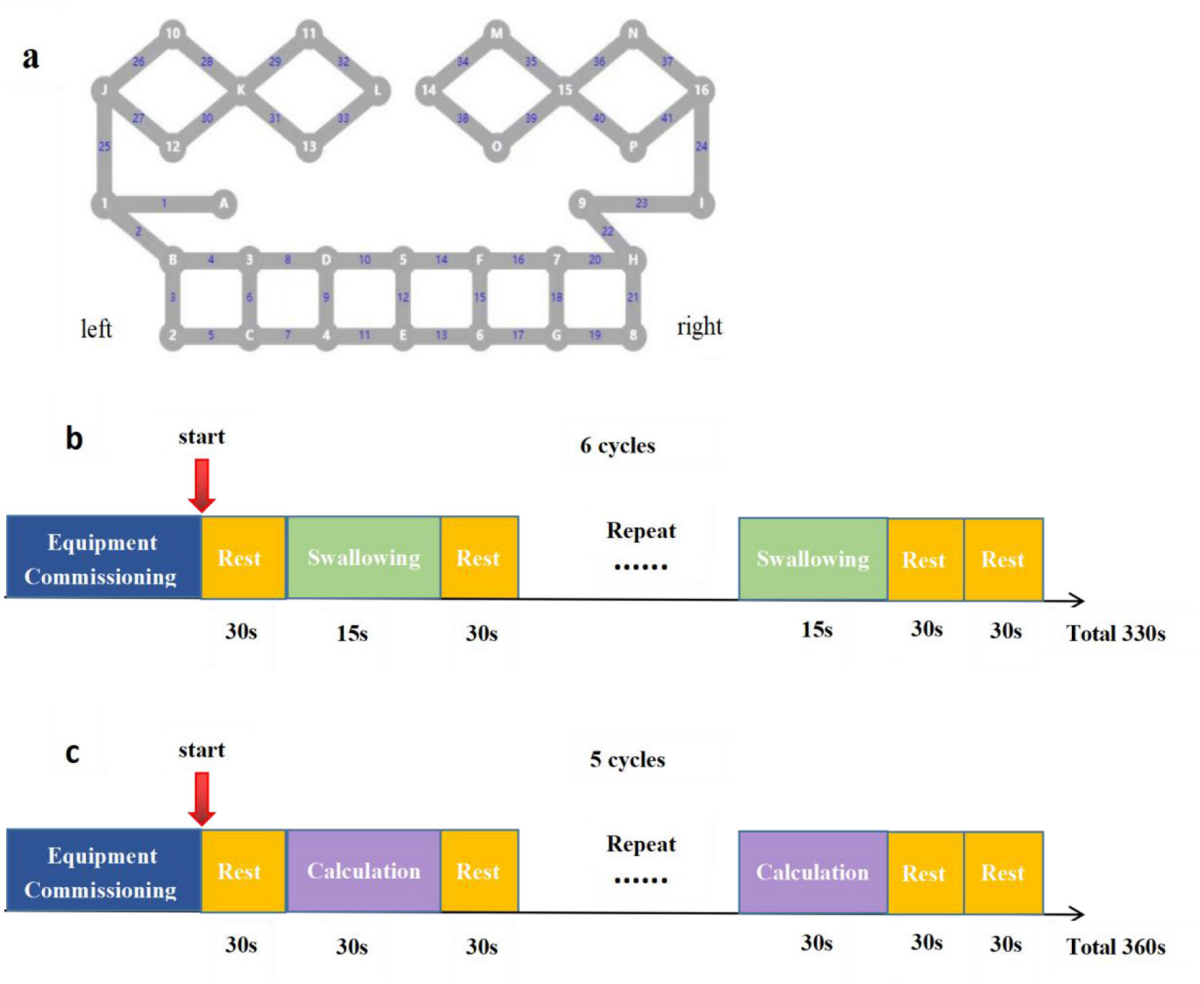
**(a)** Brain localization schema of channels. **(b)** Cognitive fNIRS protocol. **(c)** Swallowing fNIRS protocol.

We used an fNIRS system (BS-3000, Wuhan Znion Technology Co., Ltd., Wuhan, China) with wavelengths of 695 and 830 nm to acquire the data. The fNIRS cap is a device configured with 16 near-infrared light emitters and 16 detectors (spaced nearly 2 cm apart), forming a total of 41 channels and covering the prefrontal, parietal, and part of the temporal lobes (Figure 4a and Table 1). Based on the EEG-10–20 system, we normalized the wearing of the fNIRS hat, with Cz generally located between optrode L and 14 and FPz at optrode E. The fNIRS system acquired neuroimaging data at a sampling rate of 20 Hz, and the data were provided from the BS-3000 to a personal computer via the TCP/IP protocol for further online processing using MATLAB R2014.

**Table 1:** The fNIRS channels of different brain region.

**Brain region**	**fNIRS channels information**
Left DLPFC	Channels 5; Channels 6; Channels 8
Left FPA	Channels 7; Channels9; Channels10; Channels11
Left PreM&SMC	Channels 1; Channels 25; Channels 27; Channels 30; Channels 31; Channels 33
Left PMC	Channels 28; Channels 29; Channels 32; Channels 26
Left Broca	Channels 2; Channels 3; Channels 4
Right Broca	Channels 20; Channels 21; Channels 22
Right PMC	Channels 37; Channels 34; Channels 35; Channels 36
Right PreM&SMC	Channels 38; Channels 39; Channels 40; Channels 41; Channels 24; Channels 23
Right FPA	Channels 13; Channels 14; Channels 15; Channels 17
Right DLPFC	Channels 16; Channels 18; Channels 19

We prepared a quiet and tidy room in advance to minimize the interference of the external environment on the data collection process. Prior to the start of the trial, the operator was responsible for checking the operational status of the equipment and fully informing the subjects of the precautions to be taken during the data acquisition and the fNIRS design protocol. With the fNIRS cap on, the operator adjusted the position of the optical probe and ruffled the subjects' hair to ensure that the device was calibrated at 95% or higher.

For the cognitive fNIRS protocol (Figure 4b), the subjects were instructed to take a 30-s rest, then complete a cognitive task (30-s calculation and 30-s rest, repeated 5 times). The cognitive task was set to randomly present numbers between 400 and 500 and subtract 3 consecutively until the end of the task. For the swallowing fNIRS protocol (Figure 4c), the subjects took a 30-s rest, then completed the swallowing task as instructed (2–3 swallows in 15 s and a 30-s rest, repeated 6 times). Both cognitive and swallowing tasks were reminded to the patient visually through the computer screen. In the calculation of the cognitive program, the patient was required to dictate the results of the calculations, and the topic of the calculations was presented through the visual cue of the computer screen, with the tester minimizing head and neck bobbing as much as possible throughout the process. The researcher only verbally reminded the patient to take a break and stop the task at the beginning of the break in the testing process. For better visual detection of the subject's data acquisition process, the researcher and the subject were located 1 m behind the patient at the beginning of the fNIRS acquisition to monitor the process, while minimizing the impact on the subject as much as possible.

### 2.6 Mylohyoid motor-evoked potentials assessment (MH-MEP)

**Figure 5:**
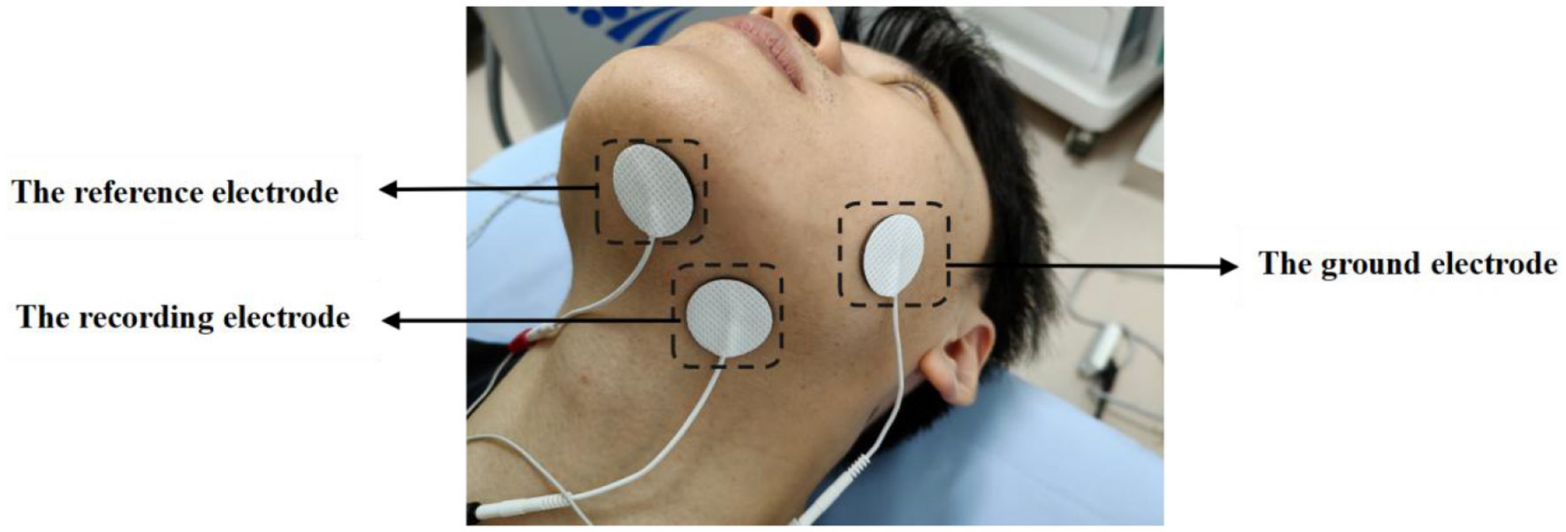
Recording site of mylohyoid motor evoked potentials.

To determine the repetitive transcranial magnetic stimulation output intensity, we measured MH-MEP (one of the measurements of swallowing-related motor-evoked potentials) before the subjects underwent the rTMS intervention. The evaluator used a single-pulse transcranial magnetic stimulation system (Model CCY-II; Wuhan Yiruide Medical Equipment Co., Ltd., Wuhan, China, YZB-20142211249) with an acquisition sensitivity set to 500 uV. Before evaluating the MH-MEP, the skin of the jaw and neck was cleansed with 75% alcohol to remove oil and increase the electrical conductivity between the skin and the electrodes. The subjects lay in a comfortable bed, with the recording electrode attached to the mylohyoid muscle belly, the reference electrode attached to the tendon of the mylohyoid muscle, and the ground electrode to the contralateral cheek (Figure 5). MH-MEP was performed at the end of the expiration in a resting state but was not recorded during swallows so as to avoid facilitation.

All stimulation was performed by a single-pulse transcranial magnetic stimulator equipped with a figure-of-eight-shaped coil (70 mm) at maximum output. The vertex of the cranium was first determined, and the coil was moved within an area of 2–4 cm anteriorly and 4–6 cm laterally (the cortical area of the mandibular hyoid muscle cortex). To obtain the optimal localization point, the tangential angle of the coil was appropriately adjusted near this area.

Once the evaluator had determined the optimal stimulation site, a series of single-pulse stimulations were performed at 80% of the maximum output intensity, with 20–30 s between each stimulation, and the output intensity was gradually reduced to determine the rMT. The resting threshold was defined as the lowest intensity, inducing an MH-MEP amplitude >50 uV on at least 5 out of 10 consecutive trials, and the recorded results were presented as a percentage of the maximum output intensity of the stimulator. Low-pass filtering was set to 0.01 Hz to correct for signal distortion due to respiration, heartbeat, and vasomotion.

### 2.7 Statistical analysis

The fNIRS raw data files were preprocessed and analyzed based on the Homer2_UI software and Metlab (R2014a). Following a file format conversion, the raw data were processed using hemodynamic response function (HRF) and wavelet-minimum description length (wavelet-MDL) analysis to detect and minimize the effects of motion artifacts and ambient light noise. Low-pass filtering set to 0.1 Hz, high-pass filtering 0.01 Hz, and a threshold of 20%. We used NIRS_KIT and IBM SPSS Statistics (version 26) to perform statistics and construct GLM models. To visualize the brain functional activation effects, the BrainNet Viewer software was used to draw topographic and 3D maps of the brain network. A paired sample *t-*test was used to calculate the difference between the pre- and post-intervention within the groups. A double sample *t-*test was used to calculate the post-intervention comparisons between the groups. Statistical significance was established at *P* < 0.05.

## 3 Results

A total of 50 healthy volunteers were included in this study to receive non-invasive vagal neuromodulation (taVNS+VNMM). None of the participants withdrew from the trial or reported adverse effects during the trial. The participants' baseline demographic characteristics are shown in Table 2. No statistical differences were observed between the two groups in terms of gender and age (*p* > 0.05).

**Table 2:** The participants' baseline demographic characteristics.

**Variable**	**Cognitive fNIRS protocol**				**Swallowing fNIRS protocol**			
	**taVNS**	**VNMM**	***F*** **/** *X* ^2^	***P*** **-value**	**taVNS**	**VNMM**	***F*** **/** *X* ^2^	***P*** **-value**
Age (years, mean ± SD)	24.82 ± 3.54	23.92 ± 2.72	0.525	0.476^a^	25.62 ± 4.77	25.33 ± 3.75	0.027	0.872^a^
Number of patients (*n*)	12	13			13	12		
Man/female	8/4	10/3	0.326	0.568^b^	9/4	8/4	0.019	0.891^b^

n, number; SD, standard deviation.

^a^P**-**values are obtained by using one-way analysis of variance.

^b^P**-**values are obtained by using Fisher exact test.

### 3.1 Effect of vagus nerve magnetic modulation and transauricular vagus nerve stimulation on brain activation: cognitive task-state fNIRS

**Figure 6:**
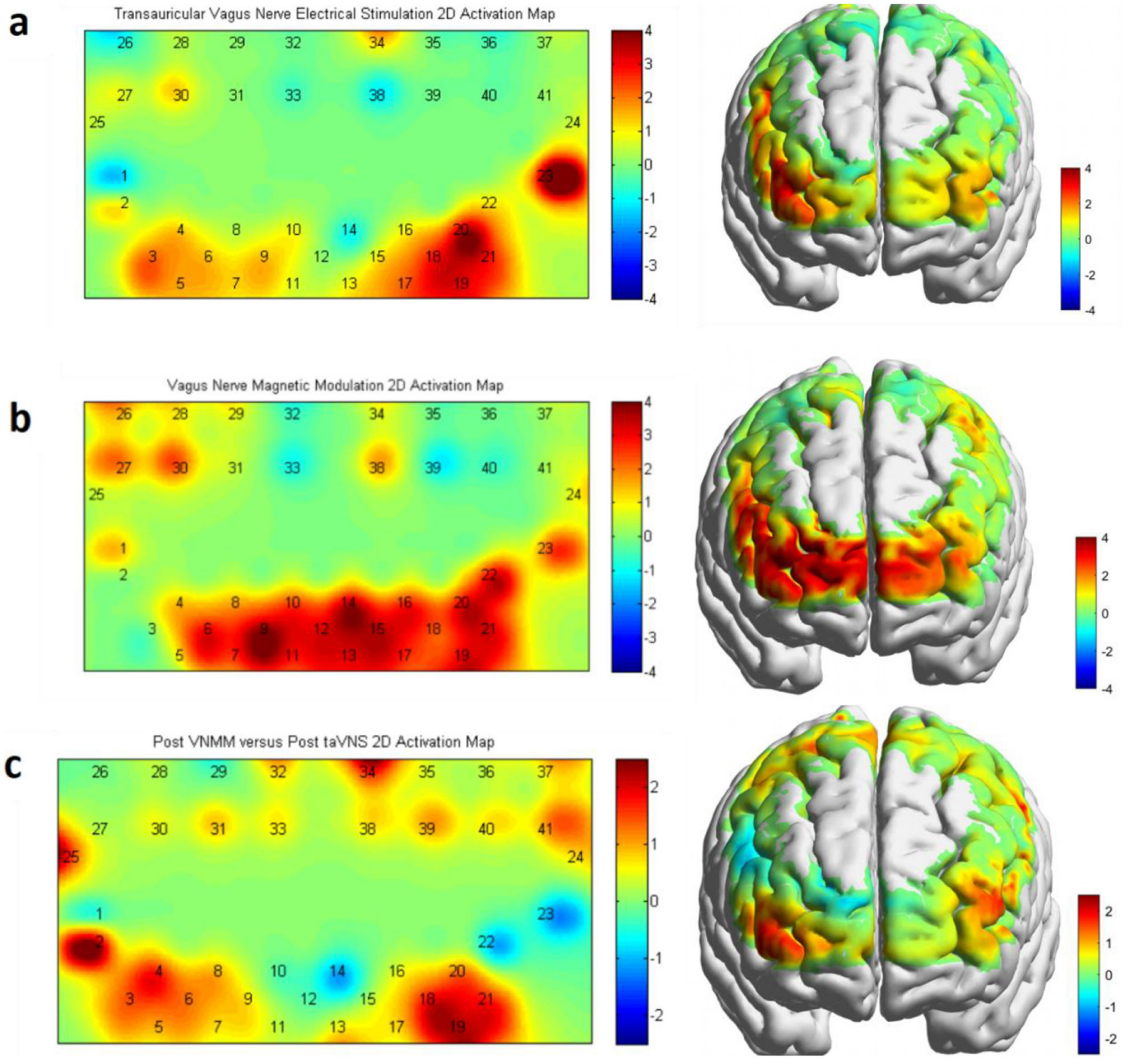
**(a)** Activation map of taVNS in a cognitive task-state fNIRS protocol. **(b)** Activation map of VNMM in a cognitive task-state fNIRS protocol. **(c)** Activation map of comparison of difference in post-intervention between taVNS and VNMM in brain activation when performing cognitive task-state fNIRS protocol.

We used cognitive task-state fNIRS to assess hemodynamic changes associated with brain activation pre- and post-VNM intervention. The fNIRS data were analyzed by a generalized linear model (GLM) to calculate β values, which reflect the strength of brain activation. Applying paired-samples *t-*tests (post- vs. pre-intervention) and FDR calibration to β values before and after the intervention within the group, we found significant differences in the taVNS group in the right dorsolateral prefrontal cortex (DLPFC) [channel 18: *P* = 0.0072, channel 19: *P* = 0.0113]; the right Broca's area [channel20: *P* = 0.0017, channel 21: *P* = 0.0301, channel 23: *P* < 0.0001]; the right prefrontal cortex (PFC) [channel 17: *P* = 0.0329]; and the left Broca's area [channel 3: *P* = 0.0389] (Figure 6a). Significant differences were found in the VNMM group in the right DLPFC [channel16: *P* = 0.0098, channel 18: *P* = 0.0481, channel 19: *P* = 0.0039], the right Broca's area [channel20: *P* = 0.0037, channel 21: *P* = 0.01, channel22: *P* = 0.0041, channel 23: *P* = 0.0192], the right PFC area [channel 13: *P* = 0.0117, channel 14: *P* = 0.0021, channel 15: *P* = 0.0039, channel 17: *P* = 0.0417], the left DLPFC [channel 6: *P* = 0.0073], the left PFC area [channel 9: *P* = 0.0011, channel 10: *P* = 0.016, channel 11: *P* = 0.0348], and the left premotor and supplementary motor areas [channel27: *P* = 0.0492, channel 30: *P* = 0.0297] (Figure 6b). Applying a double sample *t-*test (post-VNMM vs. post-taVNS) and FDR calibration to β values after the intervention between the two groups revealed significant differences in the right DLPFC [channel 18: *P* = 0.0333, channel 19: *P* = 0.0302], the right primary motor area [channel 34: *P* = 0.0148], and the left Broca's area [channel 2: *P* = 0.0091] (Figure 6c).

### 3.2 Effect of vagus nerve magnetic modulation and transauricular vagus nerve stimulation on brain activation: swallowing task-state fNIRS

**Figure 7:**
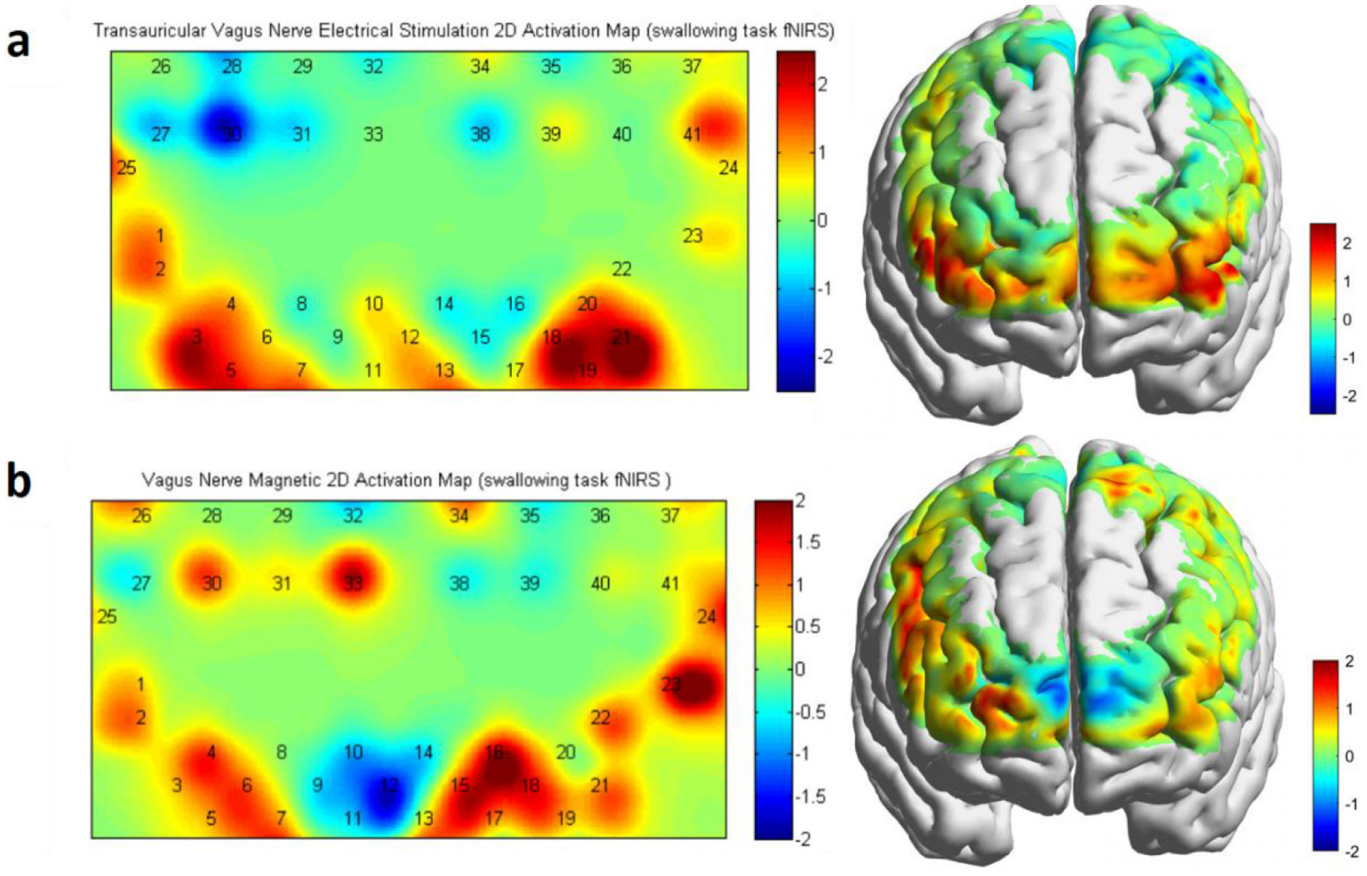
**(a)** Activation map of taVNS in a swallowing task-state fNIRS protocol. **(b)** Activation map of VNMM in a swallowing task-state fNIRS protocol.

We used swallowing task-state fNIRS to assess hemodynamic changes associated with brain activation pre- and post-VNM intervention. The fNIRS raw data files were preprocessed and analyzed based on the Homer2_UI software and Metlab (R2014a). Low-pass filtering was set to 0.01 Hz to correct for signal distortion due to respiration, heartbeat, and vasomotion. Applying paired samples *t-*tests (post vs. pre) and FDR calibration to β values before and after the intervention within the group revealed significant differences in the taVNS group in the right DLPFC [channel 18: *P* = 0.0239], the right Broca's area [channel 21: *P* = 0.0125], and the left Broca's area [channel 3: *P* = 0.035] (Figure 7a). Significant differences were found in the VNMM group in the right DLPFC [channel 16: *P* = 0.0483] and the right Broca's area [channel 23: *P* = 0.0353] (Figure 7b). Applying a double sample *t-*test (post-VNMM vs. post-taVNS) and FDR calibration to β values after the intervention between the two groups revealed significant differences in the right DLPFC [channel 16: *P* = 0.0043].

### 3.3 Effect of vagus nerve magnetic modulation and transauricular vagus nerve stimulation on brain connectivity: cognitive task-state fNIRS

**Figure 8:**
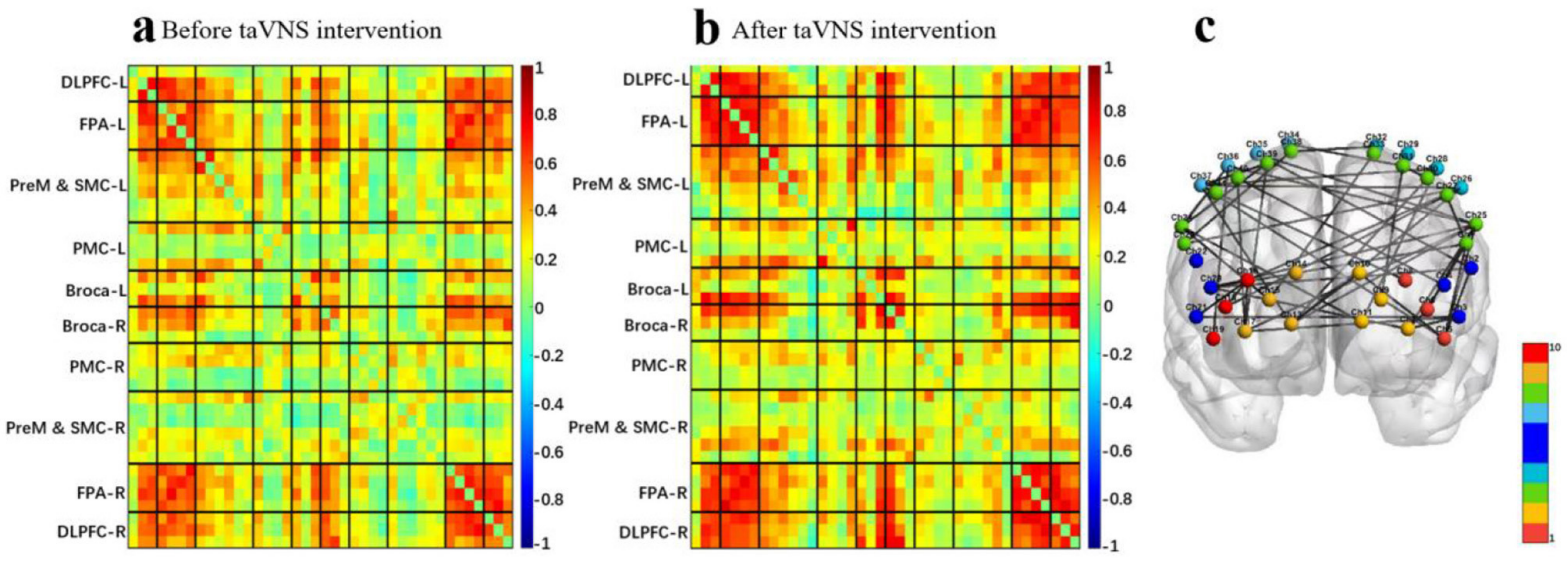
**(a)** Average functional matrix diagram of pre-taVNS intervention in a cognitive task-state fNIRS protocol. **(b)** Average functional matrix diagram of post-taVNS intervention in a cognitive task-state fNIRS protocol. **(c)** Comparison of pre- and post-taVNS intervention effective connectivity differences in a cognitive task-state fNIRS protocol.

The mean functional connectivity matrix results showed that taVNS enhanced the whole-brain mean functional connectivity matrix strength after a single intervention in the cognitive protocol (Figures 8a, b). The intra/inter-hemispheric brain network connectivity in brain regions related to cognitive tasks was also significantly enhanced. Specifically included (Figure 8c):

1) Within the left hemisphere: Broca-DLPFC [channel 2-5], Broca-FPA [channel 3-7] and DLPFC-FPA [channel 6-7].
2) Within the right hemisphere: FPA-DLPFC [channel 17-16], DLPFC-DLPFC [channel 16-18] and DLPFC-Broca [channel 16-20, channel 16-21].
3) Between left and right lateral hemispheres: left FPA-right FPA [channel 10-13], left FPA-right Broca [channel 7-20], left FPA-right PreM&SMC [channel 11-24], left Broca-right FPA [channel 3-17], left Broca-right Broca [channel 2-20, channel 3-21].

**Figure 9:**
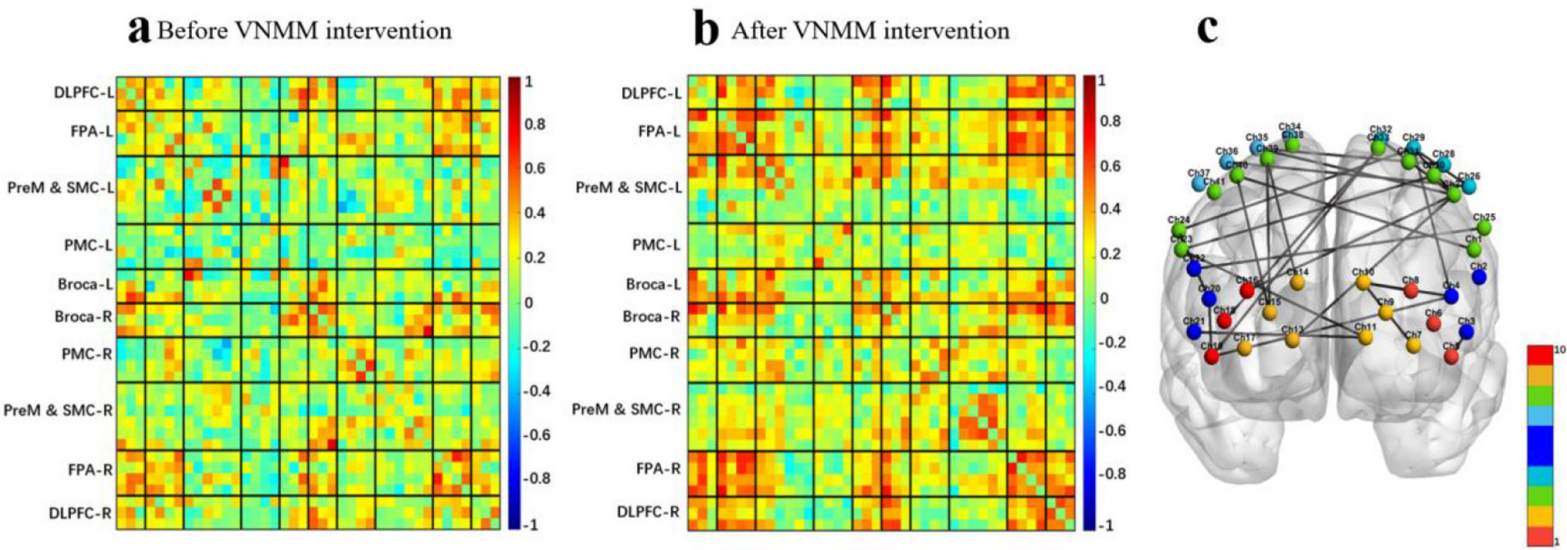
**(a)** Average functional matrix diagram of pre-VNMM intervention in a cognitive task-state fNIRS protocol. **(b)** Average functional matrix diagram of post-VNMM intervention in a cognitive task-state fNIRS protocol. **(c)** Comparison of pre- and post-VNMM intervention effective connectivity differences in a cognitive task-state fNIRS protocol.

The mean functional connectivity matrix results showed that VNMM also enhanced the whole-brain mean functional connectivity matrix strength after a single intervention in the cognitive protocol (Figures 9a, b). The intra/inter-hemispheric brain network connectivity in brain regions related to cognitive tasks was also significantly enhanced. Specifically included (Figure 9c):

1) Within the left hemisphere: Broca-DLPFC [channel 3-5], Broca-FPA [channel 4-10], FPA-FPA [channel 7-10] and DLPFC-FPA [channel 8-10].
2s) Within the right hemisphere: DLPFC-Broca [channel 19-20].
3) Between left and right lateral hemispheres: left FPA-right Broca [channel 11-21], left PreM&SMC-right FPA [channel 27-13], left PreM&SMC-right DLPFC [channel 27-16, channel 33-18, channel 33-19] and left Broca-right DLPFC [channel 4-19].

### 3.4 Effect of vagus nerve magnetic modulation and transauricular vagus nerve stimulation on brain connectivity: swallowing task-state fNIRS

**Figure 10:**
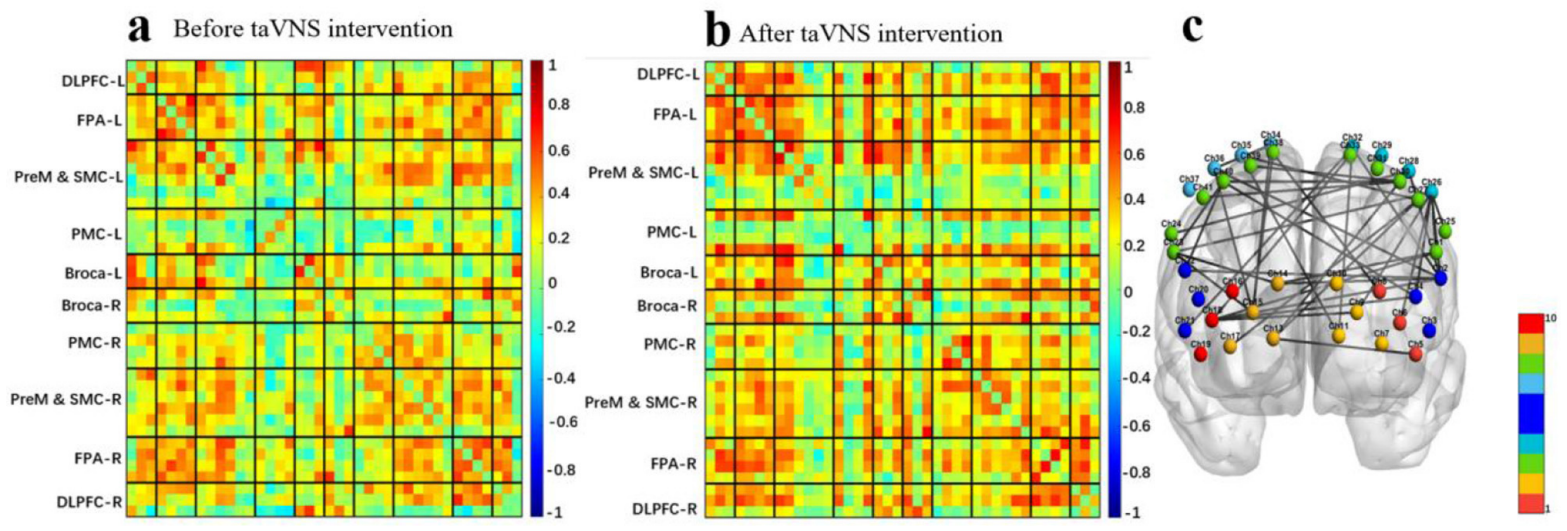
**(a)** Average functional matrix diagram of pre-taVNS intervention in a swallowing task-state fNIRS protocol. **(b)** Average functional matrix diagram of post-taVNS intervention in a swallowing task-state fNIRS protocol. **(c)** Comparison of pre- and post-taVNS intervention effective connectivity differences in a swallowing task-state fNIRS protocol.

The mean functional connectivity matrix results showed that taVNS enhanced the whole-brain mean functional connectivity matrix strength after a single intervention in the swallowing protocol (Figures 10a, b). The intra/inter-hemispheric brain network connectivity in brain regions related to swallowing tasks was significantly enhanced. Specifically included (Figure 10c):

1) Within the left hemisphere: PreM&SMC-PMC [channel 1-26, channel 1-28, channel 25-26], Broca-PMC [channel 2-26, channel 2-28, channel 2-32, channel 4-26], DLPFC-PMC [channel 6-26], FPA-PMC [channel 9-26] and FPA-PMC [channel 11-32].
2) Within the right hemisphere: FPA-PreM&SMC [channel 14-23, channel 15-23], FPA-PMC [channel 15-34, channel 15-36], PMC-PMC [channel 34-36], PMC-PreM&SMC [channel 35-38], Broca-PreM&SMC [channel 22-40] and PreM&SMC-PreM&SMC [channel 23-40].
3) Between left and right lateral hemispheres: left FPA-right DLPFC [channel 9-18], left FPA-right PMC [channel 11-35], left PreM&SMC-right FPA [channel 1-14, channel 27-13, channel 27-17, channel 30-13], left PreM&SMC-right DLPFC [channel 33-18], left PreM&SMC-right PreM&SMC [channel 30-39], left PreM&SMC-right PMC [channel 27-35], left Broca-right FPA [channel 2-14, channel 4-13], left Broca-right DLPFC [channel 2-18, channel 4-18], left Broca-right Broca [channel 2-22], left Broca-right PMC [channel 2-36], left DLPFC-right PreM&SMC [channel 8-38], left PMC-right PreM&SMC [channel 28-24] and left PMC-right Broca [channel 32-21], left DLPFC-right FPA: [channel 2-36].

**Figure 11:**
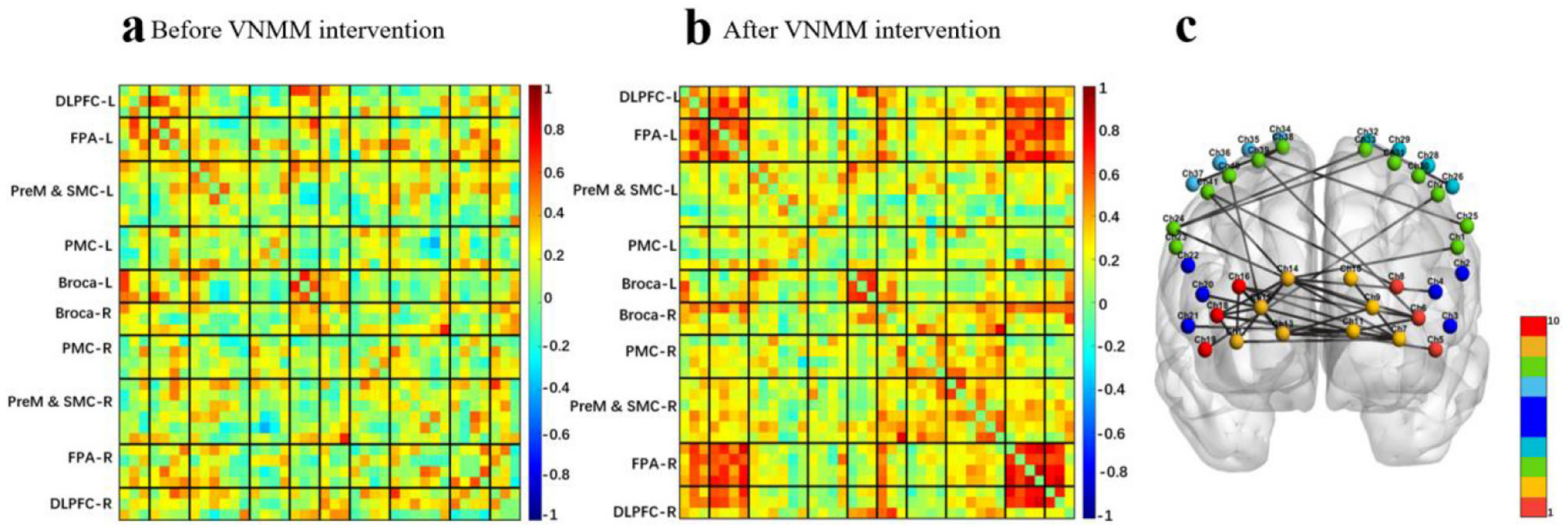
**(a)** Average functional matrix diagram of pre-VNMM intervention in a swallowing task-state fNIRS protocol. **(b)** Average functional matrix diagram of post-VNMM intervention in a swallowing task-state fNIRS protocol. **(c)** Comparison of pre- and post-VNMM intervention effective connectivity differences in a swallowing task-state fNIRS protocol.

The mean functional connectivity matrix results showed that VNMM enhanced the whole-brain mean functional connectivity matrix strength after a single intervention in the swallowing protocol (Figures 11a, b). The intra/inter-hemispheric brain network connectivity in brain regions related to swallowing tasks was also significantly enhanced. Specifically included (Figure 11c):

1) Within the left hemisphere: FPA-FPA [channel 10-7, channel 11-7], FPA- DLPFC [channel 11-6] and PMC-PMC [channel 26-32].
2) Within the right hemisphere: FPA-FPA [channel 13-15, channel 14-15, channel 14-17], FPA-DLPFC [channel 13-16, channel 14-18, channel 15-16, channel 15-18, channel 17-16, channel 17-18], FPA-Broca [channel 15-20], FPA-PreM&SMC [channel 14-24, channel 14-41, channel 15-40], PMC-PreM&SMC [channel 37-38] and PreM&SMC-PreM&SMC [channel 38-39].
3) Between left and right lateral hemispheres: left FPA-right FPA [channel 7-13, channel 7-14, channel 7-17, channel 9-13, channel 9-14, channel 11-13, channel 11-15], left FPA-right DLPFC [channel 7-16, channel 7-18], left FPA-right Broca [channel 11-21], left PreM&SMC-right FPA [channel 1-14], left PreM&SMC-right DLPFC [channel 27-19], left PreM&SMC-right PreM&SMC [channel 24-33], left PreM&SMC-right PMC [channel 25-35], left Broca-right FPA [channel 4-14], left DLPFC-right FPA [channel 6-14], left DLPFC-right DLPFC [channel 6-16, channel 5-18], left DLPFC-right PMC [channel 6-35] and left PMC-right PreM&SMC [channel 29-24].

## 4 Discussion

This study aimed to investigate the potential mechanisms and differences in the effects of taVNS and VNMM on cortical excitability in healthy individuals using a functional near-infrared swallowing and cognitive task paradigm. Overall, the cognitive and swallowing-related cortical excitability (dorsolateral prefrontal, prefrontal cortex, Broca's area, etc.) and Brain Connectivity (intra/inter-hemispheric brain) was significantly augmented in both task paradigms compared to the pre-intervention by both taVNS and VNMM. In addition, the excitatory effects mediated by taVNS and VNMM overlapped in some activation regions, and we also observed that VNMM had more significant activation effects in the cognitive and swallowing task paradigms than taVNS.

Swallowing is closely related to cognitive functioning, and the pre-initiation phase of swallowing (oral preparatory phase) is a voluntary behavior that requires recognition, matching, and judgment of the nature of the food, the use of tools, the swallowing environment, etc. However, the oral, pharyngeal, and esophageal phases may lead to changes in cognitive demands under conditions requiring voluntary enhancement of oral activity, posture, or other swallowing maneuvers (Brodsky et al., 2011). Numerous brain regions activated during swallowing are also responsible for a diverse range of cognitive functions, particularly attention and language processing (Corbetta and Shulman, 2002). A meta-analysis of studies demonstrated a significant correlation between dysphagia and cognitive impairments and that multiple neuroanatomical systems are involved in both functions simultaneously (Dehaghani et al., 2021). Ebrahimian Dehaghani et al. (2018) applied three causal models to estimate the indirect association between brain injury and dysphagia through cognition and attention and found that cognition and attention acted as mediators between brain injury and dysphagia. The complexity of swallowing combined with cognitive impairments resulting from neurological injuries may involve multiple brain regions and neural circuits, which is why clinical rehabilitation strategies for such patients need to be explored and developed. Our study found that both taVNS and VNMM significantly enhanced the excitability and connections of the brain regions related to cognitive and swallowing functions and that VNMM had more pronounced activation effects in the cognitive and swallowing task paradigms than taVNS. Given the above, vagus neuromodulation techniques may be a promising intervention for clinical patients with dysphagia combined with cognitive impairments.

Non-invasive vagus nerve neuromodulation has been approved as a more convenient and accessible neuromodulation technique for the treatment of epilepsy, depression, stroke rehabilitation, etc. Several consecutive studies with favorable results have shown that taVNS promotes quality of life, mood adjustment, and cognitive function in patients with epilepsy, depression, and stroke (Austelle et al., 2022; Harden et al., 2000; Marangell et al., 2002; Rush et al., 2005). A recent single-center randomized clinical trial reported that taVNS effectively improved cognitive function and activities of daily living compared to patients with post-stroke cognitive dysfunction who received a sham stimulation (Li et al., 2022). Zhang et al. conducted a randomized, single-blind, crossover-controlled trial to investigate the effects of taVNS stimulation on cognitive function and neural activity in the brain of healthy adults. Their results showed that VNS could lead to beneficial therapeutic effects, mainly in terms of improving memory, language skills, and attention (Zhang et al., 2022). Notably, a preliminary study of patients with long-term disorders of consciousness mediated by vagal magnetic modulation found significant improvements in the revised Coma Recovery Scale, the Glasgow Scale, and brainstem auditory-evoked potentials in patients with pDOC who were treated with VNMM—providing a basis for future research on VNMM in cognitive rehabilitation. In light of this, both techniques have promising research potential in promoting cognitive function improvement, but the underlying mechanisms and differences in their effects remain ambiguous (Wang et al., 2022a). Our study utilized functional near-infrared imaging and hemodynamic theories to elucidate the excitatory effects of taVNS and VNMM on cognitively relevant areas of the cortex. We found that taVNS and VNMM significantly activated the dorsolateral prefrontal, the prefrontal cortex, Broca's area, etc. Prefrontal cortical areas are widely connected to functional brain regions and are involved in cognitive functions such as judgment and execution, while the dorsolateral prefrontal area is predominantly associated with the sensory and motor cortex, which is key to regulating attention, thinking, and action. Furthermore, one study found that taVNS produced significant activation in the angular gyrus, caudate nucleus, cerebellum, and cingulate gyrus through fMRI; however, there was a direct effect of the differences in the stimulation parameters on BOLD signaling (Badran et al., 2018). Our findings are consistent with those that have been reported to date, which have found that at the cortical level, both taVNS and VNMM enhance intra-hemispheric and hemispheric see brain region connectivity, predominantly in the bilateral dorsolateral prefrontal, Broca's, and supplementary motor areas, among others (Cheng I. et al., 2022). It is worth mentioning that fNIRS is not able to assess subcortical areas, and thus is not able to make a holistic judgment about whole-brain changes in excitation and connectivity during task activity.

Compared to reports on the two techniques in promoting cognitive improvement, research on the application of both of them in dysphagia is still in its preliminary stages. A sham-controlled, double-blind, parallel study of patients with post-stroke dysphagia treated with VNMM showed significant improvement in swallowing outcomes at the neurophysiological, radiological, and functional dimensions (Lin et al., 2018). Wang et al. reported a case study of taVNS intervention in patients with dysphagia after dorsal lateral medullary infarction and showed that it significantly reduced salivary retention and improved swallowing incapacity (Yuan et al., 2019). Meanwhile, a recent preclinical study of taVNS treatment in rats with post-stroke dysphagia found that it significantly increased the number of swallowing times within 20 s, the expression of vascular endothelial growth factor, and basic fibroblast growth factor. Further clinical studies explored whether taVNS could promote the recovery of swallowing function in subacute post-stroke dysphagia (PSD), 40 conducted a sham-controlled, double-blind, parallel trial study randomized to receive taVNS or sham taVNS combined with routine rehabilitation training, and the results showed that ta-VNS treatment is more beneficial to improving swallowing function in patients with acute PSD (Wang Y. et al., 2022). In conjunction with the studies reported above, our study explored the effects of taVNS and VNMM on the swallowing-related cortical brain regions at the neuroimaging level and found that the subjects showed a more significant activation of the swallowing-related brain regions (DLFPC, FPC, Broca's area, etc.) after mediating the taVNS and VNMM interventions. Swallowing movements as a highly complex and coordinated behavior, both reflexive and volitional, are manifested in spatially and functionally distinct activation of the cortical and subcortical areas. Previous studies have found that during the act of swallowing saliva, the strongest region of fNIRS signaling is the inferior frontal gyrus and that repeated salivary swallowing may result in a more pronounced blood oxygenation level-dependent response than water swallowing (Kober and Hemodynamic, 2018). In addition, some scholars applied fNIRS to detect cerebral hemodynamics during volitional water swallowing and found significant increases in oxyhemoglobin concentrations in the bilateral precentral gyrus, postcentral gyrus, inferior frontal gyrus, superior temporal gyrus, middle temporal gyrus, etc (Inamoto et al., 2015).

Transcutaneous Auricular Vagus Nerve Stimulation (taVNS) and Vagus Nerve Magnetic Modulation (VNMM) are both neuromodulation techniques that target peripheral pathways by intervening at anatomical sites along the vagus nerve. Our study found that during cognitive and swallowing task paradigms, both VNMM and taVNS were capable of activating task-related brain regions with overlapping neural substrates. Moreover, VNMM appeared to exhibit more favorable stimulation effects compared to taVNS. The observed convergent or divergent effects of these two interventions warrant further investigation regarding their underlying mechanisms and potential explanations. Regarding the convergent effects observed during different task paradigms, one possible explanation is their shared activation of downstream neural pathways. taVNS targets the cutaneous branches of the vagus nerve in the auricular concha, while VNMM modulates the vagus nerve near the mastoid. Both interventions transmit sensory signals via vagal afferent fibers to the nucleus tractus solitarius (NTS) for integration and processing. Additionally, they may share common neuroplasticity regulatory mechanisms, such as long-term potentiation (LTP) or BDNF-TrkB signaling, which enhance neural plasticity in regions like the prefrontal cortex. Conversely, we propose the following hypotheses to explain the divergent effects between the two interventions. First, differences in stimulation targets and spatial precision may play a role. Although taVNS and VNMM share similar signal transduction pathways, their distinct intervention sites may lead to variations in the types (e.g., Aβ vs. C fibers) and proportions of activated nerve fibers. VNMM's magnetic field penetrates deeper tissues but with lower spatial specificity, potentially causing non-specific activation of adjacent cervical sympathetic chains. In contrast, taVNS is more superficial but susceptible to electrode placement errors. Therefore, future studies should establish finite element models based on fiber types and electric/magnetic field distributions to predict the differential activation patterns between taVNS and VNMM. Second, frequency-dependent responses may contribute to divergent effects. taVNS typically employs an alternating frequency of 20 Hz/4 Hz, whereas VNMM uses a 5 Hz stimulation frequency. The 5 Hz frequency falls within the theta band, which may facilitate coupling with hippocampal and prefrontal theta oscillations, promoting LTP and synaptic plasticity. In contrast, 20 Hz belongs to the beta band. While both 5 Hz VNMM and 20 Hz/4 Hz taVNS have been investigated independently (Park et al., 2013; Knollhoff et al., 2022), comparative analyses and mechanistic explorations remain limited. Notably, this discrepancy introduces a confounding variable that may partially compromise the equivalence of the two methods. As a feasibility study exploring the effects of dual-task vagus nerve neuromodulation using fNIRS technology, the current research has limitations, including the absence of sham stimulation controls and a small sample size, which restrict in-depth interpretation of the results. Although we observed convergent and divergent immediate effects between the two interventions, the lack of long-term randomized controlled trials and task-related behavioral data precludes definitive conclusions regarding their specific behavioral outcome.

Exploring the application of fNIRS can have important clinical implications. The technique has favorable temporal and spatial resolutions, and its portability and basic parameters may allow for the participation of people who are unavailable for other neuroimaging examinations (Yoshida et al., 2025). Regarding the types of cognitive paradigms in the context of fNIRS technology, the common ones include Go-NO-GO task, Stroop task, NO-back task, and computational task, etc. The computational task (random number consecutively minus 7), which was chosen for this study, is relatively simple compared to other cognitive task paradigms, and the researcher generally can choose the corresponding scheme according to the purpose of the study. Based on realistic clinical environments and conditions, fNIRS can support subjects undergoing longer-term monitoring and practical application in different task paradigms. These finding will assist clinical staff in arranging and guiding the rehabilitation treatment program and serve as an effective tool for adjunctive diagnostics. At the same time, fNIRS enables the joint application of multiple devices—such as functional magnetic resonance imaging, positron emission tomography, and EEG—for synchronized data acquisition and mutual corroboration.

Our data demonstrated that the activation of the cognitive and swallowing-related cortical brain regions was significantly enhanced in both taVNS and VNMM, and that there was a more obvious cortical activation effect of VNMM compared with taVNS in the cognitive and swallowing task paradigms, thereby lending new insight into the difference in effects between the two as well as options for practical clinical application. It is worth noting that this study only compared pre- and post-test measures within/between groups and did not include a sham-stimulation control group. Therefore, the observed effects could not rule out potential influences from placebo or task-specific effects. In the future, multi-center, large-sample randomized clinical controlled trials are expected to further validate the effectiveness and effect differences.

## 5 Limitations

A few study limitations are noteworthy. We only observed and analyzed the activation effects of task-state functional near-infrared imaging, which did not involve changes in functional brain connectivity between the motor and prefrontal regions. Furthermore, nodding-like swallowing movements and redundant head and neck movements may occur during repeated salivary swallowing due to lack of saliva. In addition, this study did not have a sham experimental group for control, and although the pre-intervention baseline data of the taVNS and VNMM groups were compared for calibration of the baseline situation, it was still inadequate and the results remain exploratory. Thus, the results of this study cannot exclude the occurrence of placebo or task-specific effects. Moreover, the current intensity of the taVNS stimulation we used was in the range of 5–10 mA, and although no subject has reported any significant side effect for the current, it is not certain whether the long course of intervention study is accepted as well as the safety of the study, which is worthy of further exploration. Furthermore, behavioral data were not conducted in this study at this time, and since this study was a brief one-time intervention, changes in swallowing behavioral functioning may not have been significant. Last but not least, this study was limited by the absence of sex- and age-stratified analyses and unexamined interaction effects.

## 6 Conclusion

This study demonstrates the feasibility of using fNIRS to differentiate taVNS/VNMM effects during dual-task paradigms. Preliminary data suggest VNMM may offer superior network modulation, warranting larger trials to validate behavioral correlates.

## Data Availability

The raw data supporting the conclusions of this article will be made available by the authors, without undue reservation.
